# Characterization of the chloroplast genome of *Meterostachys sikokianus* (Makino) Nakai (Crassulaceae) and its phylogenetic analysis

**DOI:** 10.1080/23802359.2021.2008837

**Published:** 2021-12-17

**Authors:** Halam Kang, Bo-Yun Kim, Ha-Rim Lee, Yoo-Jung Park, Kyung-Ah Kim, Kyeong-Sik Cheon

**Affiliations:** a Department of Biological Science, Sangji University, Wonju, South Korea; b Plant Resources Division, National Institute of Biological Resources, Incheon, South Korea; c Environmental Research Institute, Kangwon National University, Chuncheon, South Korea

**Keywords:** Crassulaceae, *Meterostachys*, chloroplast genome, phylogenetic analysis

## Abstract

*Meterostachys* is a monotypic genus of Crassulaceae, though its phylogenetic position remains unclear. Here, we report the complete chloroplast (cp) genome sequence of *Meterostachys sikokianus* using the Illumina high-throughput sequencing approach. The cp genome was 149,860 bp in length, containing a large single copy (LSC) of 82,293 bp and a small single copy (SSC) of 16,879 bp, which were separated by a pair of 25,344 bp inverted repeats (IRs). The overall GC content of the *M. sikokianus* cp genome was 37.6%. A total of 113 unique genes were annotated, consisting of 79 protein coding genes (PCGs), 30 transfer RNAs (tRNAs), and four ribosomal RNAs (rRNAs). Among these genes, eighteen contained one or two introns. A maximum-likelihood (ML) phylogenetic analysis based on 30 accessions of Crassulaceae showed that *M. sikokianus* was most closely related to *Orostachys japonica* and *Orostachys fimbriata*.

The family Crassulaceae consists of species of predominantly succulent appearance, adapted to xerophytic conditions and characterized by a specific type of crassulacean acid metabolism (CAM) (Gontcharova et al. 2006). Among these, *Meterostachys* Nakai is a monotypic genus containing only one species, *Meterostachys sikokianus* (Makino) Nakai. This was initially described as *Cotyledon sikokiana* (Makino 1891). Later, *Meterostachys* was segregated into a new genus because its morphological characteristics differed from those of *Cotyledon* (Hara 1935). However, the phylogenetic relationship and taxonomic position of *M. sikokianus* remain uncertain because allied genera such as *Hylotelephium*, *Orostachys*, and *Sinocrassula* form a polytomy according to all previous phylogenetic studies (Mort et al. 2001; Mayuzumi and Ohba 2004; Gontcharova et al. 2006; Messerschmid et al. 2020). In this study, we report the complete chloroplast (cp) genome sequence of *M. sikokianus* for the first time. This will provide useful genetic information for further studies of the phylogenetic relationships and taxonomic position of *M. sikokianus*.

The plant materials for this study were sampled from Mt. Hwaak (38°00′04′′N, 127°31′16′′E), Gapyeong-gun, Gyeonggi-do province in South Korea, and a voucher specimen was deposited at the Sangji University Herbarium (voucher no. SJUH000434; KS Cheon, cheonks@sangji.ac.kr). Total DNA was extracted using a DNeasy Plant Mini Kit (Qiagen Inc., Valencia, CA, USA). Paired-end sequencing for the cp genome of *M. sikokianus* was performed on the MiSeq (Illumina Inc., San Diego, CA, USA) platform. We obtained 3,448,502 raw reads with a length of 301 bp. The assembly and annotation of the mitogenome were accomplished using Geneious prime^®^ v.2021.1.1 (Biomatters Ltd, Auckland, New Zealand). We also compared each gene to the published complete cp genome sequence of Crassulaceae for correct gene annotation. The tRNAs were confirmed using tRNAscan-SE (Schattner et al. 2005).

A circular form of the complete cp genome of *M. sikokianus* is a DNA molecule 149,860 bp in length with 37.6 GC content, composed of a large-single-copy (LSC) region of 82,293 bp, a small-single-copy (SSC) region of 16,879 bp, and two inverted-repeat (IR) regions of 25,344 bp. The plastid genome contains a total of 113 unique genes consisting of 79 protein-coding genes, 30 transfer RNA (tRNA) genes, and four ribosomal RNA (rRNA) genes. Of which, eighteen genes are duplicated in IR regions. Additionally, two genes contain two introns (*clpP* and *ycf3*), and sixteen genes contain one intron (*atpF, ndhA*, *ndhB*, *petB*, *petD*, *rpl2*, *rpl16*, *rpoC1*, *rps12, rps16*, *trnA-UGC*, *trnG-GCC*, *trnI-GAU*, *trnK-UUU*, *trnL-UAA*, and *trnV-UAC*).[Fig F0001]

To construct the phylogenetic tree, the complete cp genomes of 29 accessions were selected within the subfamily Sempervivoideae in Crassulaceae. One additional species from the subfamily Kalanchoideae (*Kalanchoe tomentosa*) in Crassulaceae was chosen as an outgroup. The sequences were aligned using MAFFT (Katoh and Standley 2013). A maximum-likelihood (ML) analysis was performed using RAxML v.7.4.2 with 1000 bootstrap replicates and the GTR + I + Γ model (Stamatakis 2006). The ML (maximum-likelihood) tree formed three clades. The first clade consists of *Sedum* alone and formed the most basal part. The second clade is made up *Phedimus* and *Rhodiola*, with *Phedimus* forming a sister to *Rhodiola*. The last clade consists of the remaining genera excluding those mentioned above. Meanwhile, *M. sikokianus* formed a sister to *Orostachys japonica* and *Orostachys fimbriata*, and its phylogenetic position as an independent genus was supported. However, since this study was conducted with only a few taxa, it is judged that additional studies including more diverse taxa are needed.

## Data Availability

The data that support the findings of this study are openly available in GenBank of NCBI at (https://www.ncbi.nlm.nih.gov/) under the accession no. MZ365442. The associated BioProject, SRA, and Bio-Sample numbers are PRJNA722124, SRR14242008, and SAMN18746007, respectively. The ML tree for Meterostachys sikokianus based on complete chloroplast genome sequences. Kalanchoe tomentosa (MN794319) was used as outgroup. Numbers at nodes are bootstrap support values in %.
